# Nanoarchitectonics of ultrafine molybdenum carbide nanocrystals into three-dimensional nitrogen-doped carbon framework for capacitive deionization†

**DOI:** 10.1039/d4sc00971a

**Published:** 2024-06-06

**Authors:** Haolin Li, Shuaihua Zhang, Bohan Liu, Xiaoheng Li, Ningzhao Shang, Xiaoxian Zhao, Miharu Eguchi, Yusuke Yamauchi, Xingtao Xu

**Affiliations:** a Department of Chemistry, College of Science, Hebei Agricultural University Baoding 071001 Hebei China zhangshuaihua@hebau.edu.cn; b School of Advanced Science and Engineering, Waseda University Shinjuku-ku Tokyo Japan; c School of Chemical Engineering and Australian Institute for Bioengineering and Nanotechnology (AIBN), The University of Queensland Brisbane QLD 4072 Australia y.yamauchi@uq.edu.au; d Department of Materials Process Engineering, Graduate School of Engineering, Nagoya University Nagoya 464-8603 Japan; e Department of Plant & Environmental New Resources, College of Life Sciences, Kyung Hee University 1732 Deogyeong-daero, Giheung-gu Yongin-si Gyeonggi-do 17104 Korea; f Marine Science and Technology College, Zhejiang Ocean University Zhoushan 316022 Zhejiang China xingtao.xu@zjou.edu.cn

## Abstract

Molybdenum carbide (MoC) has emerged as a promising material for capacitive deionization (CDI), but the poor electrochemical kinetics in conventional MoC owing to the bulk structure and low electric conductivity limit its CDI performance. To address this challenge, herein, we develop a novel strategy to synthesize ultrafine MoC nanocrystals that are embedded within a three-dimensional nitrogen-doped carbon framework (NC/MoC). This synthesis method involves the space-confined pyrolysis of molybdate precursors within metal–organic frameworks (MOFs). In this process, molybdates are confined into the MOF crystalline structure, where MOFs provide a confined reactor and carbon source. The resulting NC/MoC with the uniformly distributed MoC nanocrystals provides sufficient active sites for the electrosorption of salt ions, while the MOF-derived NC matrix facilitates charge transfer and provides the space-confined effect for preventing the possible aggregations of MoC nanocrystals during the CDI process. The NC/MoC exhibits an impressive salt adsorption capacity (SAC, 84.2 mg g^−1^, 1.2 V), rapid desalination rate, and high cycling stability (91.4% SAC retention after 200 cycles), better than those of most previously reported carbon-based CDI materials. Besides, the possible mechanisms are systematically investigated by *ex situ* characterization and density functional theory calculations. This study opens up new avenues for the construction of metal carbide-based nanocrystals for CDI and other electrochemical applications.

## Abstract 2

Ultrafine MoC nanocrystals are confined within the MOF-derived NC matrix to generating NC/MoC. The NC/MoC based electrode achieves high CDI performance.

## Graphical abstract

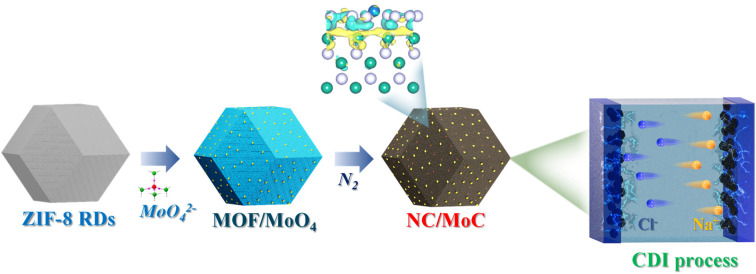

## Introduction

The worldwide water scarcity crisis, owing to the unevenly distributed freshwater, fast-growing population, or serious environmental contamination, has stimulated the urgent exploitation of highly efficient water purification/desalination techniques to meet the escalating demand for freshwater.^1–3^ Capacitive deionization (CDI), as a newly burgeoning electrochemical desalination method, has garnered considerable attention due to its high desalination efficiency, low energy consumption, and minimal secondary environmental pollution.^4–7^ As is well known, electrode materials play indispensable roles in CDI, and the developments of advanced electrode materials have been recognized as a prospective strategy for improving desalination performance.^8^ So far, various carbonaceous materials, such as activated carbon (AC),^9,10^ carbon aerogels,^11,12^ biomass-derived carbons,^13,14^ carbon nanotubes,^15^ and graphene,^16^ have been synthesized and utilized as CDI electrodes. However, the salt adsorption capacity (SAC) of most traditional carbonaceous CDI electrodes is still unsatisfactory,^17,18^ which might have originated from their deficient compositions or uncontrolled structures.^17,18^ Therefore, the carbonaceous materials should be further optimized through morphological/structural/compositional regulations, to improve their electronic conductivity, SAC as well as cycling stability for achieving large-scale practical CDI application.

Recently, molybdenum carbides (Mo_*x*_C, such as MoC or Mo_2_C) have emerged as a potentially inexpensive alternative to noble metals for electrochemical, photocatalytic, and many other applications, owing to their good electrical conductivities, tunable morphology/electronic structures, and optimal charge-transfer characteristics.^19–21^ To date, Mo_*x*_C with different crystal phases, structures, and morphologies, have been prepared using various strategies, including solid–gas reactions between molybdenum oxides and carbon–hydrogen-containing gases,^22^ solid–liquid reactions involving hydrothermal and annealing processes,^23^ solid–solid reactions *via* the carbonization of Mo-based compounds and carbon sources,^24^ chemical vapor deposition,^25^*etc*. However, the preparation of Mo_*x*_C by these previously established approaches is often performed at elevated temperatures, which easily leads to unavoidable aggregations or structural deteriorations, thereby compromising their electrochemical kinetics and further limiting their practical performance.^26^ If we develop an appropriate strategy for enhancing their structural characteristics, the targeted Mo_*x*_C will emerge as full potential candidates for high-performance CDI electrodes.^27^

**Fig. 1:**
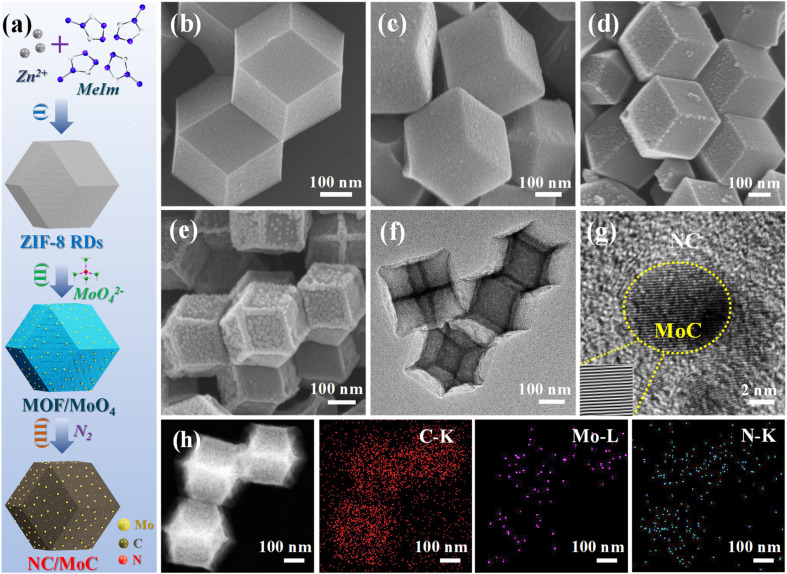
(a) Illustration of the synthetic process of NC/MoC: (I) synthesis of ZIF-8 RDs, (II) construction of MOF/MoO_4_ through functionalization of MOFs with molybdate, and (III) formation of NC/MoC by carbonization under a N_2_ atmosphere. SEM images of (b) ZIF-8 RDs, (c) MOF/MoO_4_-0.5, (d) pure ZIF-8 derived NC, and (e) NC/MoC-0.5. (f) TEM image of NC/MoC-0.5. (g) HRTEM image (Inset: the inverse fast Fourier transformation image) of NC/MoC-0.5. (h) HAADF-STEM and the corresponding element mapping images of NC/MoC-0.5.

Metal–organic frameworks (MOFs) possess highly ordered porous nanostructures with fine microstructures,^26,28–31^ which have demonstrated an emerging opportunity to serve as space-confined reactors in nanocrystal constructions.^32^ Inspired by the space-confined effect within MOFs,^26,28–31^ herein, we develop an efficient strategy to synthesize ultrafine MoC nanocrystals embedded into three-dimensional (3D) nitrogen-doped carbon (NC/MoC), through functionalization of MOFs with Na_2_MoO_4_ (denoted as MOF/MoO_4_) and a subsequent carbonization process.^26^ The detailed synthesis route of NC/MoC is schematically illustrated in Fig. 1a. First, ZIF-8 rhombic dodecahedra (RDs) were synthesized as the precursors, which were subsequently functionalized with molybdate.^33,34^ After the thermal pyrolysis of MOF/MoO_4_, the NC/MoC containing highly dispersed MoC nanocrystals confined into the N-doped porous carbon matrix was obtained. Leveraging the structural and compositional advantages, the resulting NC/MoC demonstrates promising CDI performance with large SACs (84.2 mg g^−1^ at 1.2 V; 123.4 mg g^−1^ at 1.6 V), rapid desalination rates, low specific energy consumption (0.43 W h g^−1^), and excellent long-term cycling stability (91.4% SAC retention after 200 cycles). Furthermore, the possible mechanisms were also systematically elaborated by the *ex situ* characterization and density functional theory (DFT) calculations.

## Results and discussion

The morphological evolution at each step was characterized by field-emission scanning electron microscopy (FESEM) and transmission electron microscopy (TEM). As illustrated in Fig. 1b, the initially prepared ZIF-8 RDs (average diameter, ∼500 nm) possess a well-defined rhombic dodecahedral morphology with smooth surfaces. Subsequently, a series of MOF/MoO_4_-*x* (where *x* represents the mass ratio of Na_2_MoO_4_ to ZIF-8, *x* = 0.25, 0.5, and 1) were synthesized through functionalizing MOFs with different ratios of MoO_4_^2−^. The amount of MoO_4_^2−^ was adjusted by varying the concentration of Na_2_MoO_4_ while maintaining the same quantity of ZIF-8 RDs. SEM images in Fig. 1c and S1a, b† reveal that the morphologies of MOF/MoO_4_-*x* are similar to the original rhombic dodecahedral shape of the initial ZIF-8 RDs (Fig. 1b). However, the nanocrystal sizes of MOF/MoO_4_-*x* are slightly reduced, and their rhombic edges as well as corners also become less distinct, which is consistent with previous publications.^26,35^ The as-prepared series of MOF/MoO_4_-*x* were then pyrolyzed at 800 °C under a N_2_ atmosphere; during this process, the organic ligands were converted into NC, which could further react with the adjacent Mo atoms to generate Mo_*y*_C nanocrystals (where *y* is 1 or 2),^26^ leading to the formation of NC/Mo_*y*_C heterostructures.

In contrast to the well-preserved original dodecahedral morphology of the initial ZIF-8 derived NC (Fig. 1d), the MOF/MoO_4_-*x* derived NC/Mo_*y*_C-*x* exhibit shrunk architectures with wrinkled, bumpy surfaces, and their average sizes become smaller with increasing MoO_4_^2−^ ratios (Fig. 1e, f and S1c, d†). High-resolution TEM (HRTEM) images in Fig. 1g and S2a† exhibit the emergence of the single-phase MoC nanocrystals in the NC/MoC-0.25 and NC/MoC-0.5, where MoC possesses a lattice fringe spacing of ∼0.25 nm, corresponding to the (100) plane.^26^ However, the HRTEM image in Fig. S2b† shows that the derived NC/MoC/Mo_2_C-1 possesses two lattice fringes with inter-planar spacings of 0.25 and 0.22 nm, corresponding to the (100) plane of MoC and (101) plane of Mo_2_C, respectively. Additionally, the HRTEM images in Fig. 1g and S2a† also show the presence of ultrafine MoC nanocrystals ranging from 5–10 nm in size, possibly attributed to the space-confined effect within the MOF-derived NC matrix. The high-angle annular dark-field scanning TEM (HAADF-STEM) and corresponding element mapping images in Fig. 1h reveal a homogeneous distribution of C, N, and Mo elements in NC/MoC-0.5.

**Fig. 2:**
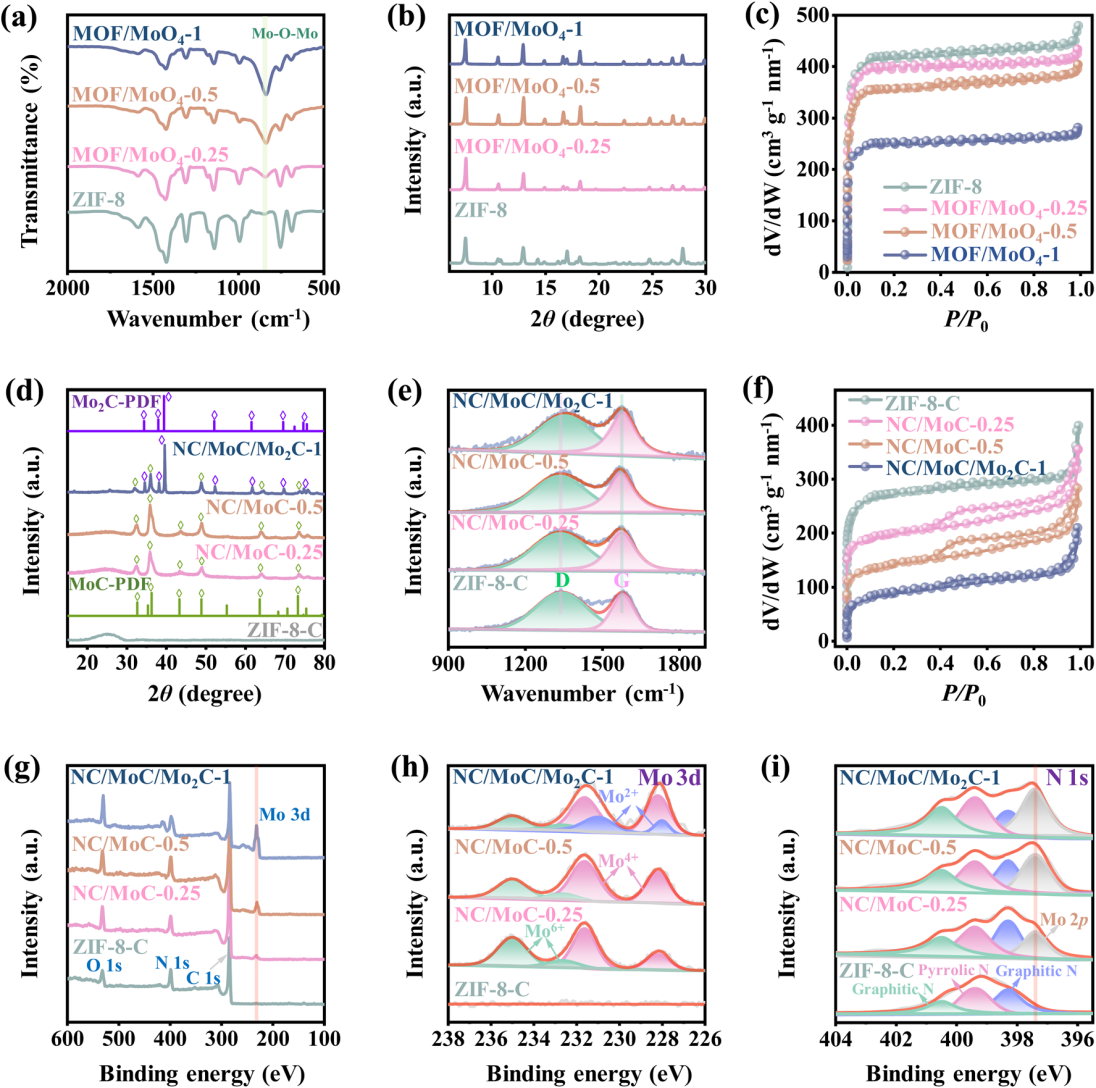
(a) FTIR spectra, (b) XRD patterns, and (c) N_2_ adsorption/desorption isotherms of the ZIF-8 RDs and MOF/MoO_4_-*x* (*x* = 0.25, 0.5 and 1). (d) PXRD patterns of MoC PDF#06-0546, Mo_2_C PDF#35-0787, ZIF-8-C, NC/MoC-0.25, NC/MoC-0.5, and NC/MoC/Mo_2_C-1. (e) Raman spectra, (f) N_2_ adsorption–desorption isotherms, (g) XPS survey spectra, and high-resolution XPS spectra of (h) Mo 3d and (i) N 1s of ZIF-8-C, NC/MoC-0.25, NC/MoC-0.5, and NC/MoC/Mo_2_C-1.

To confirm the presence of MoO_4_^2−^ units within the frameworks of ZIF-8 RDs, Fourier transform infrared spectroscopy (FTIR) spectra of MOF/MoO_4_-*x* were measured and compared with those of ZIF-8 RDs. As depicted in Fig. 2a, the FTIR spectra of the three MOF/MoO_4_-*x* maintain the main characteristic peaks of ZIF-8, but new characteristic peaks at approximately 842 cm^−1^ are observed in their FTIR spectra, assigned to the vibrations of Mo–O–Mo bonds.^36^ The intensities of these peaks gradually increase with higher proportions of MoO_4_^2−^, indicating the presence of MoO_4_^2−^ in the ZIF-8 RDs.^26^ X-ray diffraction (XRD) patterns in Fig. 2b indicate that only the ZIF-8 crystalline phase is observed in all three MOF/MoO_4_-*x*, and the addition of MoO_4_^2−^ units does not significantly affect the original crystallinity of ZIF-8.^37^ Moreover, nitrogen adsorption/desorption isotherms in Fig. 2c show that the three MOF/MoO_4_-*x* present essentially identical microporous characteristics to ZIF-8 RDs, characterized by steep N_2_ gas uptakes at low relative pressures (*P*/*P*_0_ < 0.1). The similar pore size distributions of MOF/MoO_4_-*x* and ZIF-8 RDs in Fig. S3a† further indicate that the pore structures of ZIF-8 RDs are almost not expanded or blocked after the functionalization by MoO_4_^2−^.

After pyrolysis, the MOF/MoO_4_-*x* derived NC/Mo_*y*_C-*x* with ultrafine carbide nanocrystals confined in N-doped porous carbons was obtained (Fig. 1e, f and S1c, d†).^26^ The XRD analysis in Fig. 2d reveals that only a broad diffraction peak at around 26°, corresponding to the (002) diffraction of amorphous carbon, is observed for pure ZIF-8 derived carbon (ZIF-8-C). NC/MoC-0.25 and NC/MoC-0.5 exhibit characteristic diffraction peaks at ∼32°, 36°, 43°, 49°, 63°, and 73°, indexed to the (100), (101), (103), (104), (110), and (114) diffractions of the MoC nanocrystals (JCPDS No. 06-0546), respectively. Interestingly, at a mass ratio of 1, the dual-crystalline phases of both MoC (JCPDS No. 06-0546) and Mo_2_C (JCPDS No. 35-0787) can be simultaneously recognized in the XRD pattern of NC/MoC/Mo_2_C-1, unlike the single crystal phase of MoC observed for both NC/MoC-0.25 and NC/MoC-0.5 (Fig. 2d). These XRD results, coupled with HRTEM observations, confirm that the single phase of MoC can be obtained at relatively low mass ratios (0.25 and 0.5) of MoO_4_^2−^ to MOF, while the dual-phases of MoC/Mo_2_C are obtained at a higher mass ratio of 1. Such single or dual-phases of carbides prepared through the regulation of the mass ratios have been also observed in other carbides,^38,39^*e.g.*, WC/W_2_C,^26^ Fe_2.2_C/Fe_5_C_2_,^38^ or Fe_2_C/Fe_5_C_2_/Fe_3_C.^39^

Raman spectra in Fig. 2e illustrate that two distinct peaks at approximately 1341 and 1580 cm^−1^ are observed for the three NC/Mo_*y*_C-*x* and ZIF-8-C, corresponding to the D-band associated with lattice defects, and the G-band related to the stretching vibrations of graphitic carbons, respectively.^40^ The *I*_D_/*I*_G_ ratio of NC/MoC-*x*, as the evaluation for the degrees of carbon disorders/defects, are calculated to be 1.49 (NC/MoC-0.25), 1.43 (NC/MoC-0.5), and 1.37 (NC/MoC/Mo_2_C-1), lower than that of ZIF-8-C (2.13). The *I*_D_/*I*_G_ ratio achieves a decreasing trend along with the increase in Mo_*y*_C proportions in NC/Mo_*y*_C-*x*, suggesting a decrease in lattice defects with the improved graphitization degree from NC/MoC-0.25 to NC/MoC/Mo_2_C-1, which might be beneficial for accelerating charge transfer and stabilizing the carbide nanocrystals.^26^ Nitrogen adsorption–desorption isotherms in Fig. 2f further reveal the coexistence of micropores and mesopores in the three NC/Mo_*y*_C-*x*, with the sharp nitrogen gas adsorption at low relative pressure (*P/P*_0_ < 0.1) and the hysteresis loops at higher relative pressures (∼0.4 to 1.0). Pore size distributions in Fig. S3b† also illustrate the hierarchical micro-mesoporous structures in the three NC/Mo_*y*_C-*x*, which are different from the microporous characteristics of ZIF-8-C. The specific surface areas of NC/MoC-0.25, NC/MoC-0.5, and NC/MoC/Mo_2_C-1 are 627.3, 493.9, and 308.2 m^2^ g^−1^, respectively. Furthermore, the inductively coupled plasma optical emission spectrometry (ICP-OES) determinations clearly illustrate that the contents of the Mo element exhibits an increasing trend from 38.2 wt% of NC/MoC-0.25 to 67.1 wt% of NC/MoC/Mo_2_C-1, with the improvements of the MoO_4_^2−^ units in ZIF-8 RDs (Fig. S4†).

The surface elemental compositions and valence states of the NC/Mo_*y*_C-*x* heterostructures were investigated using X-ray photoelectron spectroscopy (XPS) analysis. The survey spectra in Fig. 2g evidence the co-existence of C, N, and O elements, with the absence of Mo in ZIF-8-C. Differently, the Mo 3d peak appears in the survey XPS spectra of NC/MoC-0.25, NC/MoC-0.5, and NC/MoC/Mo_2_C-1, and its content gradually increases along with the increase in Mo_*y*_C ratios (Fig. 2g). The high-resolution XPS spectra of the Mo 3d regions (Fig. 2h) reveal two main characteristic pairs of peaks for both NC/MoC-0.25 and NC/MoC-0.5, where a pair of peaks at lower binding energies (228.9 eV for Mo 3d_5/2_ and 232.3 eV for Mo 3d_3/2_) are indexed to Mo(iv) (Mo–Mo or Mo–C bonds), while a pair of peaks at higher binding energies (233.5 eV for Mo 3d_5/2_ and 235.7 eV for Mo 3d_3/2_) are assigned to Mo(vi) (Mo–O bond) owing to the possible surface oxidation of NC/Mo_*y*_C-*x*.^26,41,42^ With regards to NC/MoC/Mo_2_C-1, another pair of peaks at binding energies of 228.8 eV (Mo 3d_5/2_) and 231.6 eV (Mo 3d_3/2_) are also obviously observed, originating from Mo(ii) of Mo_2_C.^42^ The high-resolution N 1s spectra of ZIF-8-C and NC/Mo_*y*_C-*x* (Fig. 2i) can be deconvoluted into three peaks at 398.3, 399.4, and 400.6 eV, corresponding to pyridinic-N, pyrrolic-N and graphitic-N, respectively. The additional Mo 2p characteristic peaks can also be observed in the XPS spectra of NC/MoC-0.25, NC/MoC-0.5, and NC/MoC/Mo_2_C-1.

**Fig. 3:**
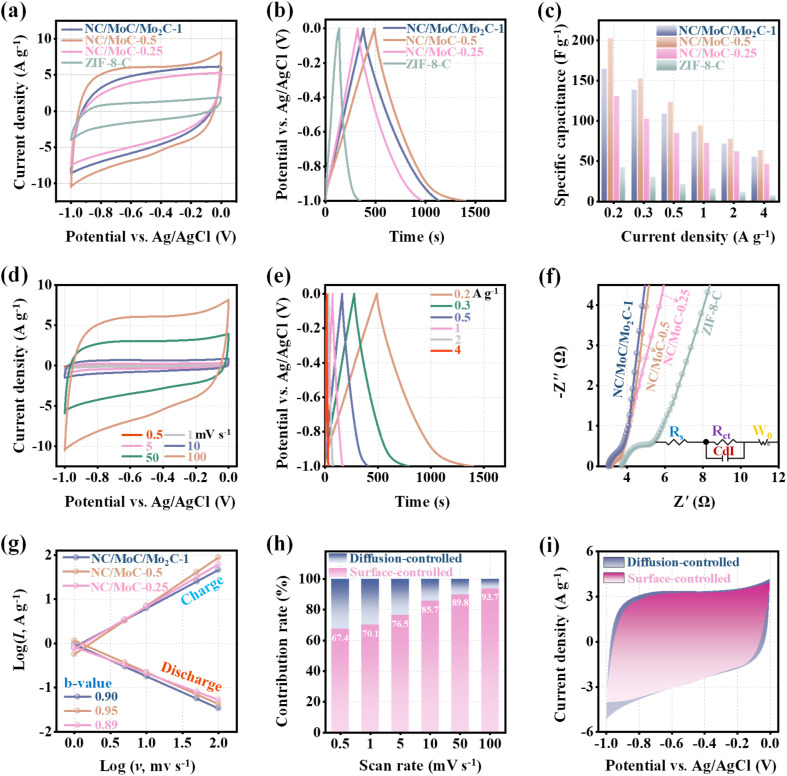
(a) CV curves at 100 mV s^−1^, (b) GCD curves at 0.2 A g^−1^, and (c) the specific capacitance values at different current densities for NC/MoC-0.25, NC/MoC-0.5, NC/MoC/Mo_2_C-1, and ZIF-8-C. (d) CV curves of NC/MoC-0.5 at the scan rates from 0.5 to 100 mV s^−1^. (e) GCD curves of NC/MoC-0.5 at current densities from 0.2 to 4 A g^−1^. (f) Nyquist plots of the three NC/Mo_*y*_C-*x* and ZIF-8-C. Inset: the equivalent circuit diagram of NC/MoC-0.5. (g) The fitted curves between current densities and the scan rates in charge and discharge processes for NC/MoC-0.25, NC/MoC-0.5 and NC/MoC/Mo_2_C-1. (h) Normalized proportions of surface- and diffusion-controlled contributions at different scan rates for NC/MoC-0.5. (i) Decoupling of surface- and diffusion-controlled contributions for NC/MoC-0.5 at a scan rate of 50 mV s^−1^.

The electrochemical performance of NC/MoC-0.25, NC/MoC-0.5, NC/MoC/Mo_2_C-1, and ZIF-8-C electrodes was systematically evaluated by cyclic voltammetry (CV) and galvanostatic charge/discharge (GCD) in 1.0 M NaCl solution. All CV curves of NC/Mo_*y*_C-*x* and ZIF-8-C exhibit nearly symmetrical rectangular shapes without any additional redox peaks at 100 mV s^−1^ (Fig. 3a), indicating the capacitive behaviors for ion storage.^43^ The three NC/Mo_*y*_C-*x* electrodes achieve significantly larger integral areas of CV curves than ZIF-8-C, and in particular, NC/MoC-0.5 exhibits the largest integral area among these electrodes, which shows its highest specific capacitance. GCD measurements were conducted to explore the charge storage capacity of these electrodes at a current density of 0.2 A g^−1^ over the potential range of −1.0 to 0 V (Fig. 3b). The NC/Mo_*y*_C-*x* electrodes display approximately symmetrical triangular shapes, with NC/MoC-0.5 also achieving the longest discharge time and therefore the highest specific capacitance^43^ (202.4 F g^−1^) compared to the other electrodes. The detailed specific capacitances of these electrodes at different current densities are further illustrated in Fig. 3c, which suggests the highest specific capacitance of NC/MoC-0.5 across all ranges of current densities. Furthermore, Fig. 3d reveals the corresponding CV profiles of the NC/MoC-0.5 electrodes recorded at scan rates ranging from 0.5 to 100 mV s^−1^. The CV integral area of NC/MoC-0.5 gradually increases with higher scan rates, indicating the boosted capacitive performance of NC/MoC-0.5. The GCD curves of NC/MoC-0.5 in Fig. 3e maintain relatively symmetrical triangular shapes with the reduction of current densities ranging from 4 to 0.2 A g^−1^, which indicates the rapid and reversible ion/charge storage processes even at low current densities. Similar tendencies are observed in the CV and GCD curves of ZIF-8-C, NC/MoC-0.25, and NC/MoC/Mo_2_C-1 (Fig. S5–S7†). The Nyquist plots of NC/Mo_*y*_C-*x* and ZIF-8-C in Fig. 3f exhibit quasi-semicircles in the high-frequency region and straight segments in the low-frequency region. Notably, the three NC/Mo_*y*_C-*x* electrodes possess smaller intersection values with the *X*-coordinates compared to ZIF-8-C, illustrating their alleviated charge-transfer impedance.^17^ Additionally, a simulated equivalent circuit (inset of Fig. 3f) suggests that the calculated charge transfer resistance (*R*_ct_) value of NC/MoC-0.5 (0.12 Ω) is obviously lower than that of ZIF-8-C (1.01 Ω), indicating the enhanced charge transfer kinetics of NC/MoC-0.5 during Na^+^ reversible desalination/regeneration.

To gain further insight into the electrochemical kinetics of NC/Mo_*y*_C-*x*, their surface-controlled (*e.g.*, capacitive contribution) and diffusion-controlled processes (*i.e.*, redox contribution) were calculated according to eqn (2) and (3) (S1.6†).^40,44,45^ As illustrated in Fig. 3g, the fitted curves between the logarithm of current densities (log *I*) and the logarithm of scan rates (log *v*) are straight lines for both the discharging and charging steps. Their corresponding *b* values range from 0.89 to 0.95, suggesting that the capacitive contributions of NC/Mo_*y*_C-*x* are mainly governed by the surface-controlled process rather than the diffusion-controlled process.^18^ The detailed contributions of surface and diffusion behaviors of NC/MoC-0.5 were calculated, and Fig. 3h manifests that the surface-contributions are gradually increased with the enhancements of the scan rates from 0.5 to 100 mV s^−1^. At a scan rate of 50 mV s^−1^, the contributions of surface-controlled and diffusion-controlled processes are determined to be 89.8% and 10.2%, respectively (Fig. 3i). These electrochemical measurements and analyses demonstrate that NC/MoC-0.5 exhibits a rapid and reversible ion storage process with capacitive characteristics, highlighting its significant potential for CDI application.

**Fig. 4:**
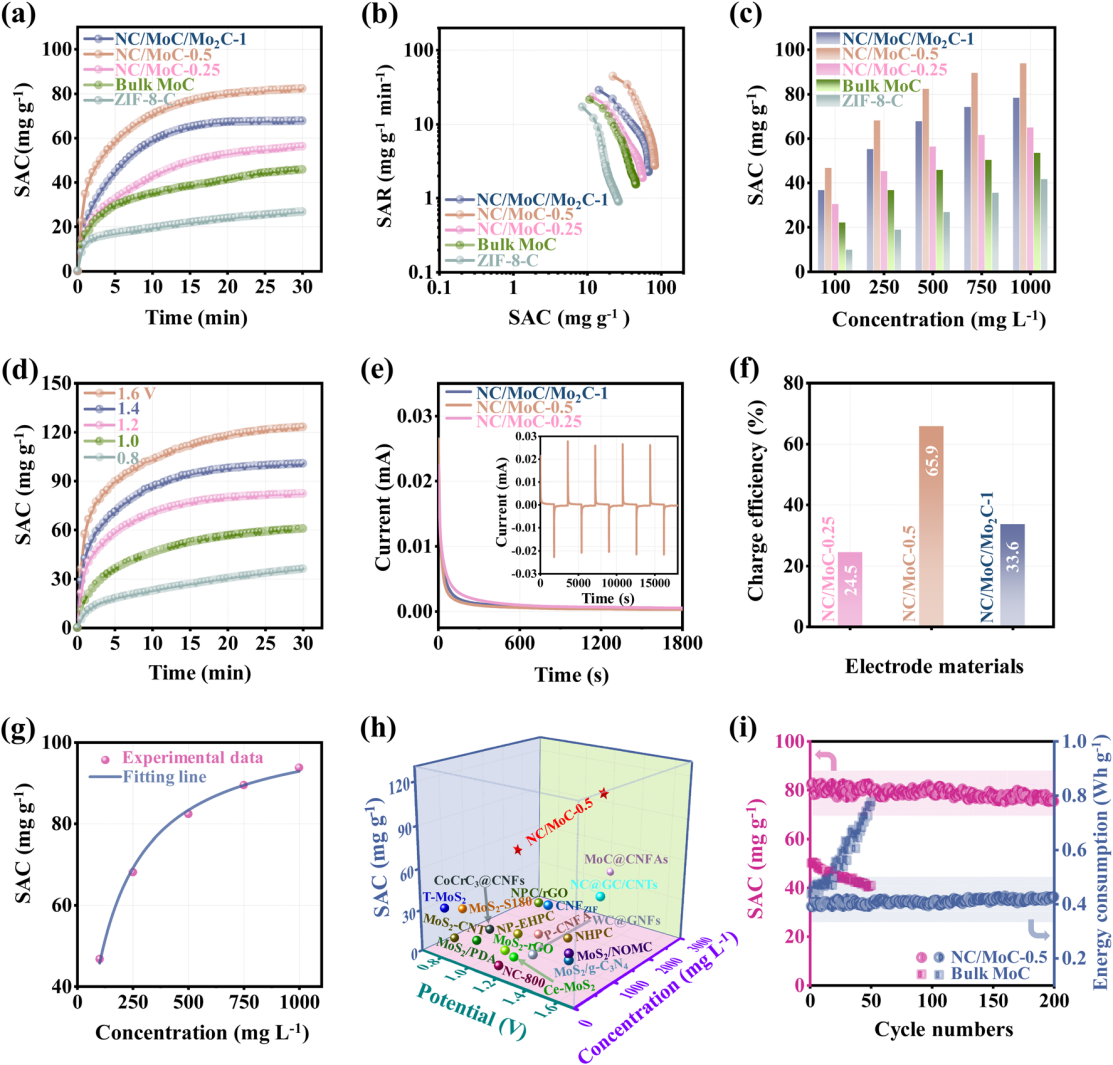
(a) Dynamic SAC *versus* deionization time plots, and (b) corresponding CDI Ragone plots of the three NC/Mo_*y*_C-*x*, bulk MoC, and ZIF-8-C in 500 mg L^−1^ NaCl solution at 1.2 V. (c) SAC values at different NaCl concentrations from 100 to 1000 mg L^−1^ for the three NC/Mo_*y*_C-*x*, bulk MoC, and ZIF-8-C (applied voltage: 1.2 V). (d) Dynamic SAC *versus* deionization time plots of NC/MoC-0.5 with an initial concentration of 500 mg L^−1^ using different voltages from 0.8 to 1.6 V. (e) Current response curves of the three NC/Mo_*y*_C-*x*, and the corresponding current variation of NC/MoC-0.5 during the desalination/regeneration cycle (inset). (f) The calculated charge efficiencies of the three NC/Mo_*y*_C-*x*. (g) The fitted isotherm adsorption curves of the Langmuir model (applied voltage: 1.2 V; NaCl concentration: from 100 to 1000 mg L^−1^). (h) Comparison of CDI performance between NC/MoC-0.5 and some reported transition metal carbides/dichalcogenides or MOF-derived carbons. (i) The recycling performance of bulk MoC and NC/MoC-0.5, along with their corresponding energy consumption changes over 200 cycles (applied voltage: 1.2 V; NaCl concentration: 500 mg L^−1^).

The exceptional electrochemical activities of the NC/Mo_*y*_C-*x* electrodes are used to investigate their CDI applications. CDI experiments were carried out in a symmetric CDI cell^18^ consisting of the NC/Mo_*y*_C-*x* electrodes as both the cathode and anode. For comparison, a bulk MoC and ZIF-8-C based CDI cells were also assembled. Desalination experiments were conducted in a 500 mg L^−1^ NaCl solution at an applied voltage of 1.2 V. The dynamic curves of the calculated SAC values (eqn (5), S1.7†) *versus* deionization time in Fig. 4a reveal that the SAC values of these electrodes increase quickly in the initial period, indicating the rapid capture of Na^+^ and Cl^−^. As deionization time progresses, the SAC values gradually increase and reach equilibrium. It is worth noting that NC/MoC-0.5 achieves an ultrahigh SAC value of 82.4 mg g^−1^, markedly exceeding those of ZIF-8-C (26.7 mg g^−1^), bulk MoC (45.8 mg g^−1^), NC/MoC-0.25 (56.3 mg g^−1^) and NC/MoC/Mo_2_C-1 (67.8 mg g^−1^). The CDI Ragone plots were used to assess the deionization performance of NC/Mo_*y*_C-*x*, bulk MoC and ZIF-8-C, including the SAC and salt adsorption rate (SAR, eqn (6), S1.7†). As illustrated in Fig. 4b, the NC/MoC-0.5 electrode occupies the more right and upper region, indicating its larger SAC and faster SAR among these electrodes. Furthermore, the effects of the initial NaCl concentrations and working voltages on CDI performance were further verified. Fig. 4c shows that the SAC values of NC/Mo_*y*_C-*x*, bulk MoC and ZIF-8-C show a gradual improvement with increasing NaCl concentrations. More importantly, NC/MoC-0.5 acquires superior SAC values compared to ZIF-8-C, bulk MoC, NC/MoC-0.25 and NC/MoC/Mo_2_C-1 across all concentration ranges from 100 to 1000 mg L^−1^, reaching a large SAC value of 93.8 mg g^−1^ at a concentration of 1000 mg L^−1^. With the extension of working voltages, both the SAC and SAR values of NC/Mo_*y*_C-*x*, and ZIF-8-C manifest pronounced improvements with increasing voltages from 0.8 to 1.6 V (Fig. 4d and S8, S9†), and the CDI Ragone plots shift to the upper-right domain at higher voltages, attributed to the reinforced electrosorption of ions (Na^+^ and Cl^−^) onto the electrodes driven by accelerated electrostatic force.^17^

The real-time current responses of the NC/Mo_*y*_C-*x* electrodes were also simultaneously monitored during the CDI process (Fig. 4e). These current changes originate from the cycling desalination/regeneration of Na^+^ and Cl^−^ by the NC/Mo_*y*_C-*x* electrodes at the applied voltage^46,47^ (inset of Fig. 4e). The charging efficiency (*Λ*) values are calculated to be 24.5%, 65.9%, and 33.6% (eqn (7), S1.7†) for NC/MoC-0.25, NC/MoC-0.5, and NC/MoC/Mo_2_C-1, respectively (Fig. 4f). The higher charge efficiency of NC/MoC-0.5 indicates its lower energy consumption during the CDI desalination process.^46,47^ And the energy consumption of NC/MoC-0.5 was determined to be 0.43 W h g^−1^ (eqn (8), S1.7†),^17^ significantly lower than that of NC/MoC-0.25 (1.13 W h g^−1^) and NC/MoC/Mo_2_C-1 (1.08 W h g^−1^). Additionally, the Langmuir isotherm model (eqn (9), S1.7†) has been adopted to simulate the changes in experimental SACs for NC/MoC-0.5 as a function of the initial NaCl concentrations ranging from 100 to 1000 mg L^−1^. As illustrated in Fig. 4g, the correlation coefficient *r*^2^ is calculated to be 0.996, revealing that the Langmuir isotherm matches well with the experimental SACs. The maximum theoretical SAC (*q*_m_) of NC/MoC-0.5 predicted from the Langmuir isotherm is 104.9 mg g^−1^.

In view of the aforementioned experimental results and discussions, the newly synthesized metal carbide, NC/MoC-0.5, exhibits several superior characteristics such as a high SAC and rapid salt adsorption rate, which evidently exceeded those of Mo-based composites like transition metal carbides/dichalcogenides, or MOF-derived carbons (Fig. 4h and Table S1†). Furthermore, the cycling stability, also termed as long-term desalination/regeneration performance, was also experimentally measured using an initial NaCl concentration of 500 mg L^−1^ at 1.2 V. Fig. 4i shows that the bulk MoC exhibits unsatisfactory SAC degradation and an increase in energy consumption only after 50 cycles. In contrast, the NC/MoC-0.5 electrode achieves a high SAC retention rate of ∼91.4% after 200 cycles, without pronounced enhancements in energy consumption throughout the long-term CDI cycles. Additionally, the SEM, TEM, HRTRM images, and XRD patterns in Fig. S10† after 200 CDI cycles demonstrate the well-maintained morphology and crystal structure of NC/MoC-0.5, further illustrating its outstanding structural stability during the consecutive CDI deionization/regeneration process, which agrees well with the above CDI cycling results (Fig. 4i). The good cycling stability of NC/MoC-0.5 might be attributed to the evenly distributed MoC nanocrystals confined in the NC matrix, and the space-confined effect might efficiently avoid the possible aggregations during the long-term CDI process.

**Fig. 5:**
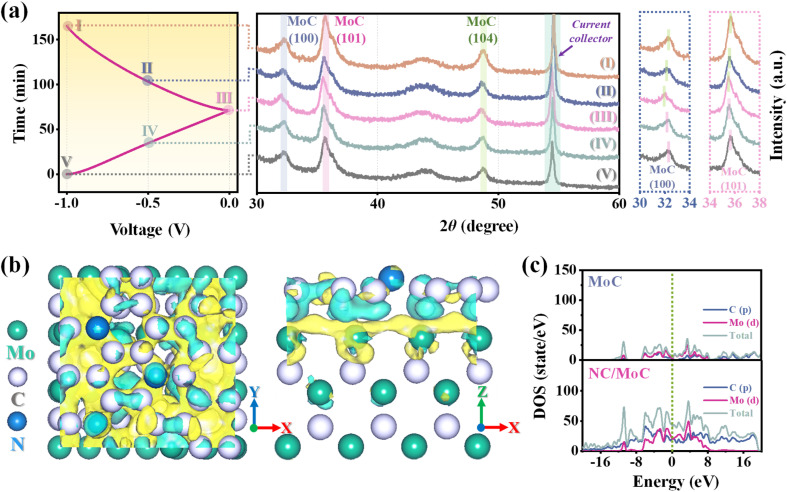
(a) The charge/discharge curve and corresponding *ex situ* XRD patterns of NC/MoC-0.5 in various desalination/regeneration stages. (b) Differential charge density of the NC/MoC, where the reduction and increase in electron density are marked by green and yellow colors, respectively. (c) Electron density of states (DOS) of the NC/MoC and pure MoC.

To further understand the possible desalination mechanism, *ex situ* XRD characterization studies^48–50^ were performed for the NC/MoC electrode at various applied voltages, corresponding to the desalination/regeneration process of the CDI measurements. As shown in Fig. 5a, some typical diffraction peaks of the (100), (101), and (104) planes from the NC/MoC-0.5 shift to lower 2*θ* angles during the desalination process (step I to III), and subsequently return back to higher original 2*θ* angles after the regeneration of the NC/MoC-0.5 electrode (step III to V). Such behaviors might originate from the reversible insertion/extraction of Na ions^46,51^ into the frameworks of MoC crystals,^52^ thereby bringing out an increase or a decrease in the crystal lattice parameters. Furthermore, DFT calculations were conducted to understand the high CDI performance of NC/MoC (S2†), with the structural models of MoC, NC, and NC/MoC depicted in Fig. S11.† The calculated differential charge density of NC/MoC in Fig. 5b shows that the charge accumulates near the surface of MoC and depletes near the NC surface, revealing the transfer of electrons from NC to the neighboring MoC, and therefore generating interfacial electronic coupling^53,54^ between the NC and MoC interfaces. The charge accumulation at the heterointerface is supposed to be beneficial to facilitate electron transport during the desalination/regeneration process.^53,54^ The density of states (DOS) in Fig. 5c also illustrates an obvious enhancement in charge density at the Fermi level for NC/MoC compared with pure MoC, which manifests the higher electrical conductivity of NC/MoC for boosted CDI performance.

## Conclusion

In summary, we have successfully synthesized MoC nanocrystals embedded into MOF-derived NC *via* functionalization of MOFs with molybdates and a subsequent thermal carbonization strategy. Due to the unique architecture of NC/MoC, the uniformly distributed MoC nanocrystals were steadily confined within the NC matrix, therefore achieving a large salt adsorption capacity (84.2 mg g^−1^, 1.2 V; 123.4 mg g^−1^, 1.6 V), fast desalination rate, and good cycling stability over 200 cycles. DFT calculations and *ex situ* XRD characterization further elucidated that the MoC nanocrystals confined in the NC matrix could facilitate electron transport during the desalination/regeneration process, thereby enhancing the CDI performance compared with pure MoC. These experimental and theoretical insights provide valuable guidance and inspiration for the design and fabrication of carbide-based nanocrystals, opening new avenues for CDI applications, and potentially extending to other applications such as electrochemical adsorption and energy storage conversion.

## Data availability

All the data supporting this article have been included in the main text and the ESI.†

## Author contributions

H. L. and S. Z. carried out the experiments, analyzed the data, and wrote the manuscript. B. L. and X. L. carried out the experiments. N. S., X. Z., and M. E. assisted in the analysis, investigation and validation. S. Z., Y. Y., and X. X. designed the project, provided the funding acquisition, analyzed the data, and revised the manuscript.

## Conflicts of interest

There are no conflicts to declare.

## Supplementary Material

SC-015-D4SC00971A-s001
