# Supplementary material for: Nanoarchitectonics of ultrafine molybdenum carbide nanocrystals into three-dimensional nitrogen-doped carbon framework for capacitive deionization
Source: Chem Sci. 2024 Jun 6;15(29):11540–9. doi: 10.1039/d4sc00971a (PMC11268501; doi:10.1039/d4sc00971a)
Supplement: SC-015-D4SC00971A-s001 [file SC-015-D4SC00971A-s001.pdf]

Electronic Supplementary Material (ESI) for Chemical Science.
This journal is © The Royal Society of Chemistry 2024

S1. Experimental Section

S1.1. Chemicals

Methanol (≥ 99.5%), *N*, *N*-Dimethylformamide (DMF, ≥ 99.8%), isopropanol (≥ 99.5%), and *N*-methyl-2-pyrrolidone (NMP, 99.9%) were obtained from Makclin Chemistry (Shanghai, China). Zinc acetate dihydrate ($Zn(OAc)_2 \cdot 2H_2O$, 99%), 2-methylimidazole (MeIm, 98%) and sodium molybdate (Na_2MoO_4, 99%) were purchased from Aladdin Chemistry (Shanghai, China). Polyvinylidene fluoride (PVDF 6020), carbon black (Super P), and Nafion solution (5 *wt.*%) were provided by Sigma-Aldrich (Merck, Shanghai, China). Bulk molybdenum carbide (MoC, 99.95%) for the comparison was obtained from Aladdin Chemistry (Shanghai, China). All chemicals were used as purchased without further processing.

S1.2. Construction of ZIF-8 RDs

For a typical synthesis of ZIF-8 RDs [S1, S2], 1.0 mmol (219.5 mg) of $Zn(OAc)_2 \cdot 2H_2O$ was dissolved into 10 mL of ultrapure water to form a homogeneous Zn^{2+} solution. 10 mmol (821.1 mg) of MeIm was dissolved into 10 mL of ultrapure water to prepare another solution. The Zn^{2+} solution was quickly added into the MeIm solution. After vigorously stirring for 5 min, the mixture solution was kept at room temperature for 24 h to obtain the white precipitates. The white precipitates were separated by centrifugation and thoroughly washed with water and methanol for several times, and dried in vacuum oven at 60 °C overnight to harvest ZIF-8 RDs.

S1.3. Preparation of MOF/MoO_4-x

MOF/MoO_4-*x* (*x* stands for the mass ratio of Na_2MoO_4 to ZIF-8, *x*=0.25, 0.5, and 1) were prepared according to the previous report [S1] with some modifications. Briefly, 200 mg of the ZIF-8 RDs were ultrasonically dissolved into 20 mL of DMF. Following this, 100 mg of Na_2MoO_4 with the mass ratio of 0.5 was added into the above ZIF-8 RDs solution to synthesis MOF/MoO_4-0.5. Under stirring for 20 min, the mixture solution was transferred into a Teflon-lined stainless steel autoclave and kept at 150 °C for 6 h. After cooling down to ambient temperature, the MOF/MoO_4-0.5 was collected by centrifugation and washed by DMF and methanol for several times, and finally dried in vacuum oven.

Another two amounts of Na_2MoO_4 (50 and 200 mg) with the mass ratios of 0.25 and 1 were also added into the ZIF-8 RDs solution (200 mg in DMF) for the construction of MOF/MoO_4-0.25 and MOF/MoO_4-1 by using the similar procedures as described above, respectively.

S1.4. Synthesis of NC/Mo$_y$C-x

The above MOF/MoO_4-x precursors were placed into a ceramic boat and thermally carbonized under nitrogen gas flow in a tube furnace, yielding NC/Mo$_y$C-x (y represents the atomic ratio of Mo to C, y= 1 or 2; x stands for the mass ratio of Na_2MoO_4 to ZIF-8, x=0.25, 0.5, and 1). During the carbonization process, the MOF/MoO_4-x were firstly heated from room temperature to 400 °C with a heating rate of 2 °C min^{-1} and keep for 3 h. Subsequently, the temperature was further improved from 400 to 800 °C with the same heating rate (2 °C min^{-1}) and also kept for another 3 h. After cooling down to room temperature, the NC/Mo$_y$C-x products were obtained. Specifically, the MOF/MoO_4-0.25 and MOF/MoO_4-0.5 precursors were converted into NC/MoC-0.25 and NC/MoC-0.5 with the single-phased MoC nanocrystals, while the MOF/MoO_4-1 precursor was transformed to the dual-phased NC/MoC/Mo_2C-1 after the thermal pyrolysis [S1-S3]. For comparison, ZIF-8 RDs were pyrolyzed to yield the carbonaceous materials, denoted as ZIF-8-C.

S1.5. Characterizations

The structure and morphology of the MOF/MoO_4-x and NC/Mo$_y$C-x were observed by field emission scanning electron microscopy (SEM, Hitachi SU-8000, Japan) with an accelerating voltage of 10.0 kV. Transmission electron microscopy (TEM), high-resolution TEM (HRTEM) images, and elemental mapping analysis were performed using a JEM-2100F (JEOL, Japan) operated at 200 kV. X-ray photoelectron spectroscopy (XPS) spectra were collected on a PHI Quantera SXM (ULVAC-PHI, Japan) instrument with an Al Kα radiation, and the binding energies were calibrated by referencing them to the C 1s (284.5 eV) binding energy. X-ray diffraction (XRD) patterns were examined by using a Rigaku Rint 2000 X-ray diffractometer (Tokyo, Japan) with monochromatic Cu Kα radiation (40 kV, 40 mA) at a scanning rate of 2° min^{-1}. Nitrogen adsorption-desorption isotherms were obtained by using an Autosorb-iQ Automated Gas Sorption System

(Quantachrome, USA) at 77 K. The specific surface area was evaluated by the multipoint Brunauer-Emmett-Teller (BET) method based on the adsorption data. The pore-size distributions were calculated from the adsorption branches of isotherms based on the density functional theory method. Fourier-transformed infrared spectra (FT-IR) were obtained using a Bruker Alpha spectrometer (Ettlingen, Germany) in the range of 500-2000 cm^{-1}. The concentration of NaCl solution was continuously measured by a REX DDSJ-308F conductivity meter (INESA Scientific Instrument, Shanghai, China).

S1.6. Electrocatalysis measurements

All electrochemical measurements were carried out in 1 M NaCl aqueous electrolyte at room temperature with a three-electrode system on Chenhua CHI 760E electrochemical workstation (Shanghai, China). The Pt/C electrode and KCl-saturated Ag/AgCl were adopted as counter electrode and reference electrode, respectively. The working electrode slurry was prepared by ultrasonically dispersing 2 mg of as-synthesized samples in a mixed solution consisting of 950 μL of isopropanol and 50 μL of Nafion solution (5.0 *wt.*%).

The specific capacitances (C, F g^{-1}) are calculated by the following eqn [1] from the GCD curves [S4, S5]:

$$C = \frac{I \times \Delta t}{m \times V} \quad [1]$$

where I is the current (A), Δt is the discharge time (s), m is the mass of sample (g), and V is the voltage window (V).

The correlation between the measured current (i) and the scanning rate (v) can be expressed by the following eqn [2] and [3]:

$$i = av^{b} \quad [2]$$

$$\log i = b\log v + \log a \quad [3]$$

where the modulatory parameters a and b, obtained from the fitted curves, can indicate whether the process is diffusion-controlled (when b approaches 0.5) or capacitive-controlled (when b is close to 1.0) [S6, S7].

The contribution mechanisms of various charging and discharging processes are calculated by the following eqn [4] [S6, S7]:

$$i(V) = k_1 v + k_2 v^{1/2} \quad [4]$$

where $k_1 v$ and $k_2 v^{1/2}$ represent the capacitive-controlled contribution and diffusion-controlled contribution, respectively.

S1.7. Desalination performance measurements

The CDI measurements were carried out in a continuous cycle system including a peristaltic pump, constant current power supply, stirring device, a tank and a CDI unit consisting of a pair of working electrodes (2.5 ×2.5 cm^2) and a pair of ion exchange membranes. The CDI electrodes were fabricated by a slurry mixing in NMP solution of the NC/Mo_yC-x: carbon black: PVDF=8: 1: 1. The slurry was coated on graphite paper (2.5 ×2.5 cm^2) and dried overnight at 80 ºC in a vacuum. In the CDI experiments, the ion conductivity meter was used to monitor and measure the real-time change of NaCl concentration at the outlet of CDI device under different concentrations (100, 250, 500, 750, and 1000 mg L^{-1}) and voltages (0.8, 1.0, 1.2, 1.4, and 1.6 V). The volume of NaCl solution was 32 mL, and the flow rate was 20 mL min^{-1}. The salt adsorption capacity (SAC, mg g^{-1}) and salt adsorption rate (SAR, mg g^{-1} min^{-1}) at t min are calculated as following eqn [5] and [6]:

$$\mathrm{SAC} = \frac{(C_0 - C_t) \times V_s}{m} \quad [5]$$

$$\mathrm{SAR} = \frac{\mathrm{SAC}}{t} \quad [6]$$

where C_0 and C_t are the NaCl concentrations at initial stage and t min (mg L^{-1}), respectively; V_s is the solution volume (L); and m is the total mass (16 mg) of the electrode materials on the working electrodes [S8, S9].

The charge efficiency (Λ) is calculated according to eqn [7] [S10, S11]:

$$\Lambda = \frac{\Gamma \times F}{\Sigma} \quad [7]$$

where Γ represents the desalination capacity (mol g^{-1}), F denotes the Faraday constant (96485 C mol^{-1}), and Σ stands for the integral of the current-time curve (C g^{-1}).

The energy consumption (E, Wh g^{-1}) is obtained according to eqn [8] [S10, S11]:

$$E = \frac{v \times \int idt}{3.6 \times (C_0 - C)\, V} \qquad [8]$$

where the v indicated a driven potential, $\int idt$ corresponded to the integrated value of the current transient *vs.* running time plot, and V (mL) was the rotational solution volume. C_0 and C (mg L^{-1}) were initial and final concentrations, respectively.

The Langmuir isotherm model (eqn [9]) is adopted to simulate the experimental SAC data of ion adsorption on the electrode [S12, S13]:

$$q = \frac{q_m K_L C}{1 + K_L C} \qquad [9]$$

where q is the SAC (mg g^{-1}), K_L is the Langmuir constant related to the heat of adsorption, q_m is the maximum SAC (mg g^{-1}) corresponding to complete monolayer coverage, and C is the equilibrium concentration (mg L^{-1}).

S2. DFT Calculation Methods

The Cambridge sequential total energy package (CASTEP) module in DFT-based Material Studio was utilized to perform all calculations [S14, S15]. Perdew-Burke-Ernzerhof (PBE) within the generalized gradient approximation (GGA) is employed to calculate the exchange-correlation potential. The cutoff energy is set to be 400 eV. All structures were optimized until the energy converged to 1.0×10^{-5} eV and the force reached 0.02 eV Å^{-1}.

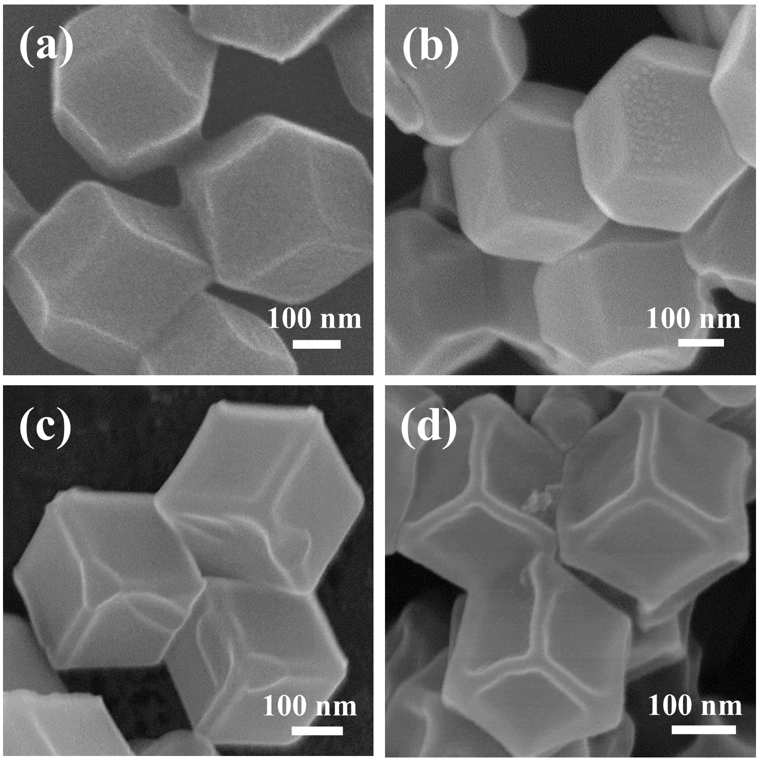

Fig. S1. SEM images of (a) MOF/MoO_4-0.25, (b) MOF/MoO_4-1, and their-derived (c) NC/MoC-0.25, and (d) NC/MoC/Mo_2C-1.

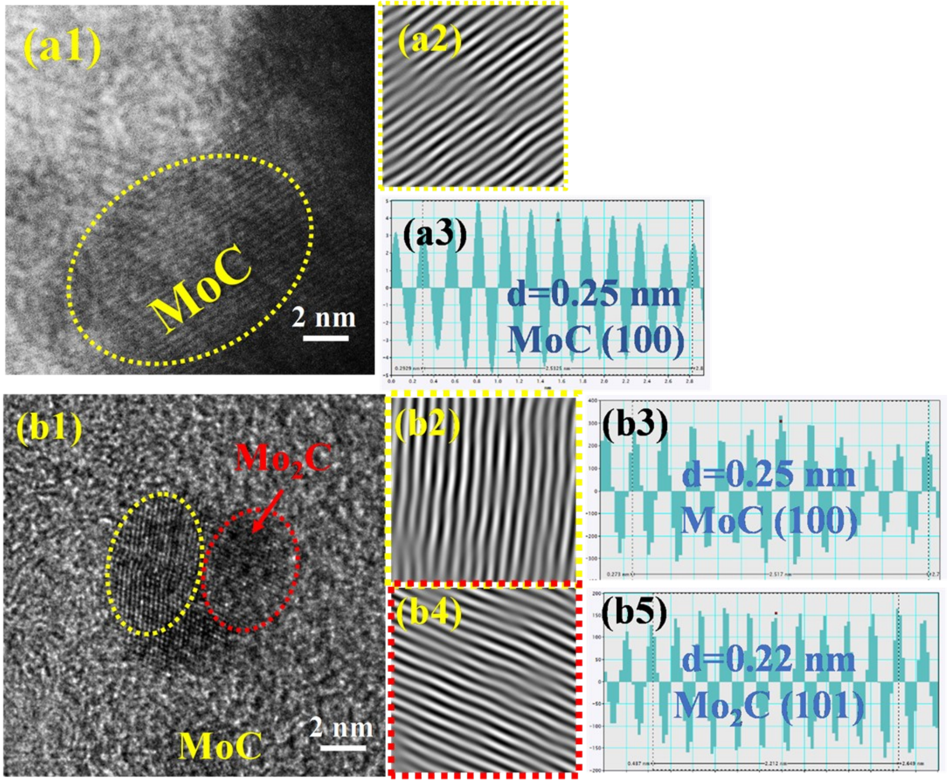

Fig. S2. (a1) HRTEM, (a2) inverse fast Fourier transformation (IFFT) image, and (a3) corresponding line scan of NC/MoC-0.25. (b1) HRTEM, and (b2-b4) IFFT images and corresponding line scans of NC/MoC/Mo_2C-1, including (b2, b4) IFFT images and (b3, b5) corresponding line scans.

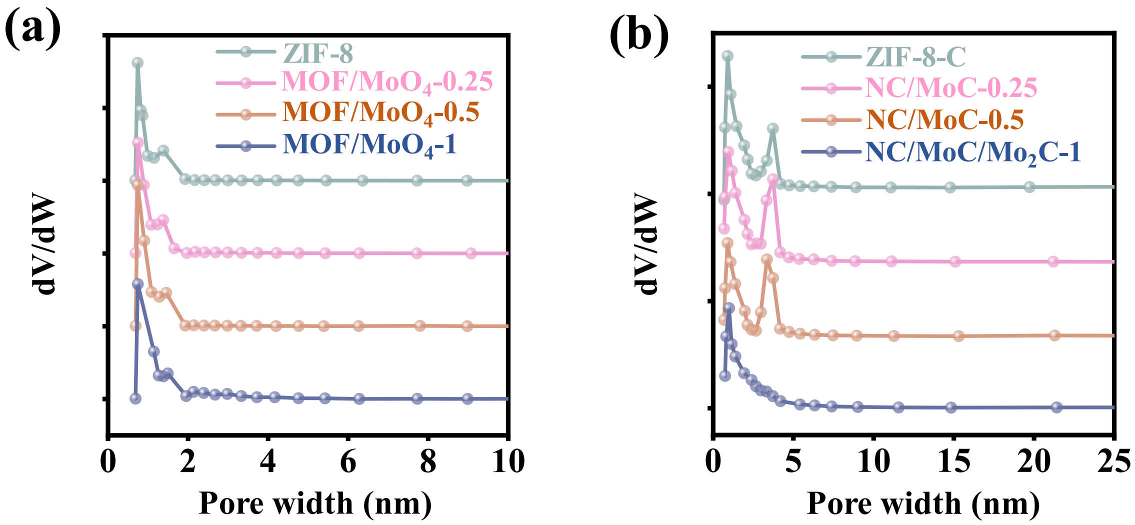

Fig. S3. Pore size distributions of (a) ZIF-8 RDs and MOF/MoO_4-*x* (*x*=0.25, 0.5, and 1) and (b) their derived ZIF-8-C, NC/MoC-0.25, NC/MoC-0.5, and $NC/MoC/Mo_2C$-1.

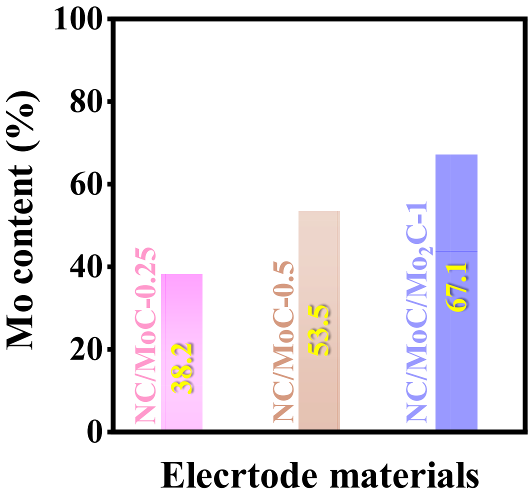

Fig. S4. The Mo contents in NC/MoC-0.25, NC/MoC-0.5, and NC/MoC/Mo_2C-1.

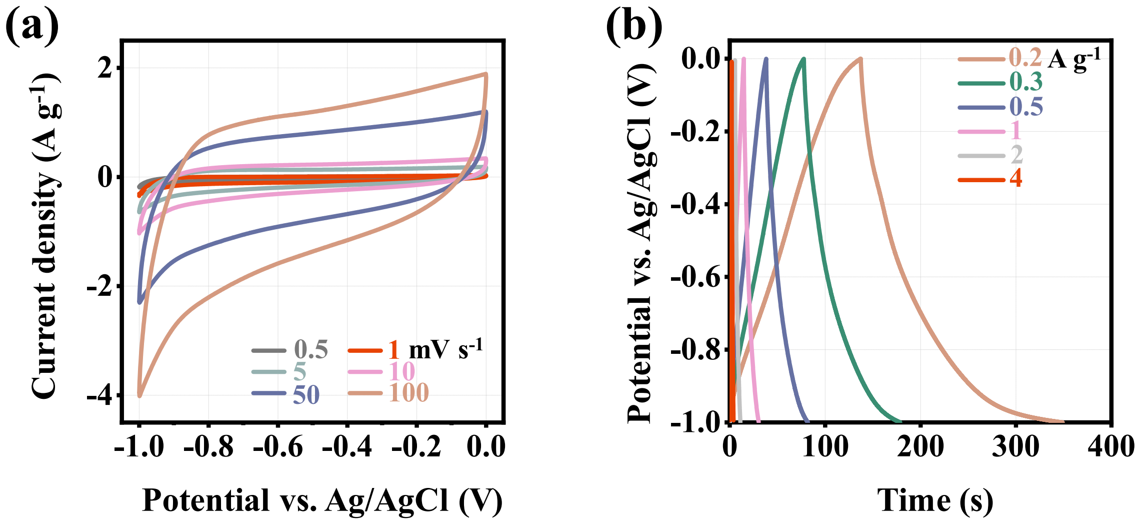

Fig. S5. (a) CV and (b) GCD curves for ZIF-8-C.

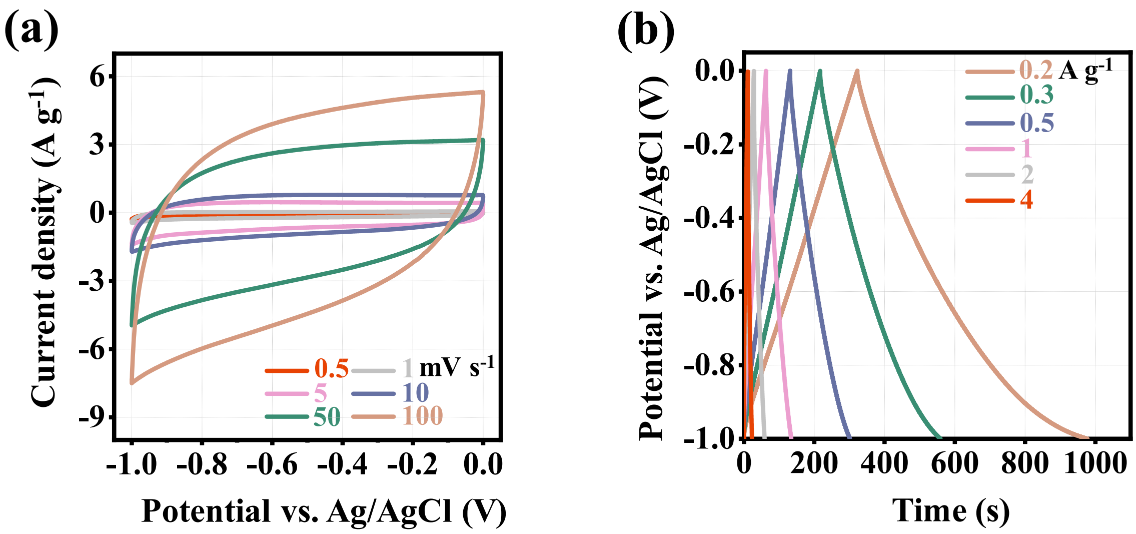

Fig. S6. (a) CV and (b) GCD curves of NC/MoC-0.25.

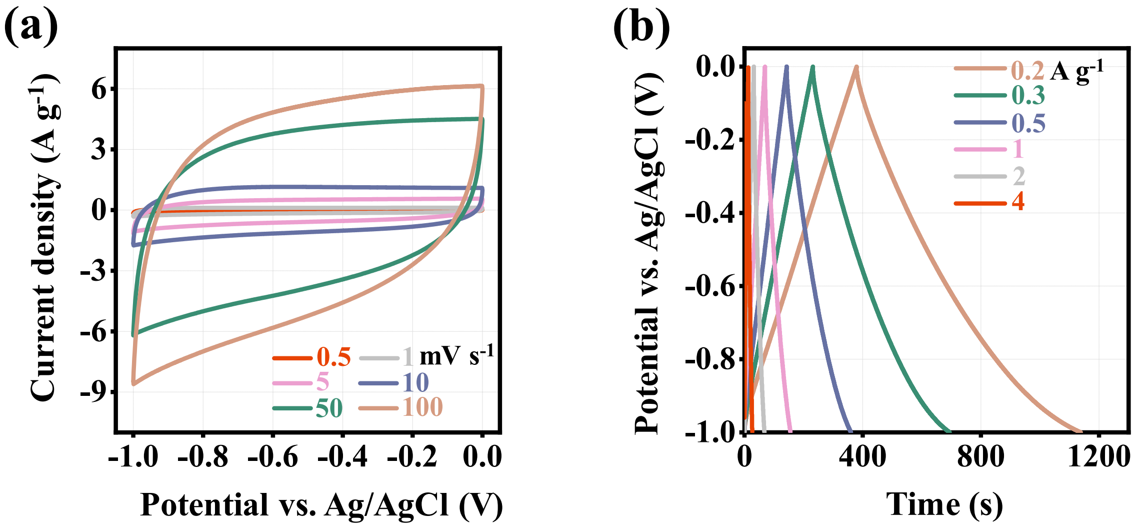

Fig. S7. (a) CV and (b) GCD curves of NC/MoC/Mo_2C-1.

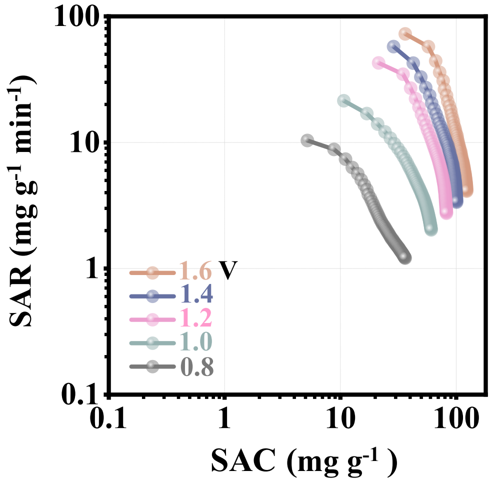

Fig. S8. CDI Ragone plots of NC/MoC-0.5 with an initial NaCl concentration of 500 mg L^{-1} at various voltages from 0.8 to 1.6 V.

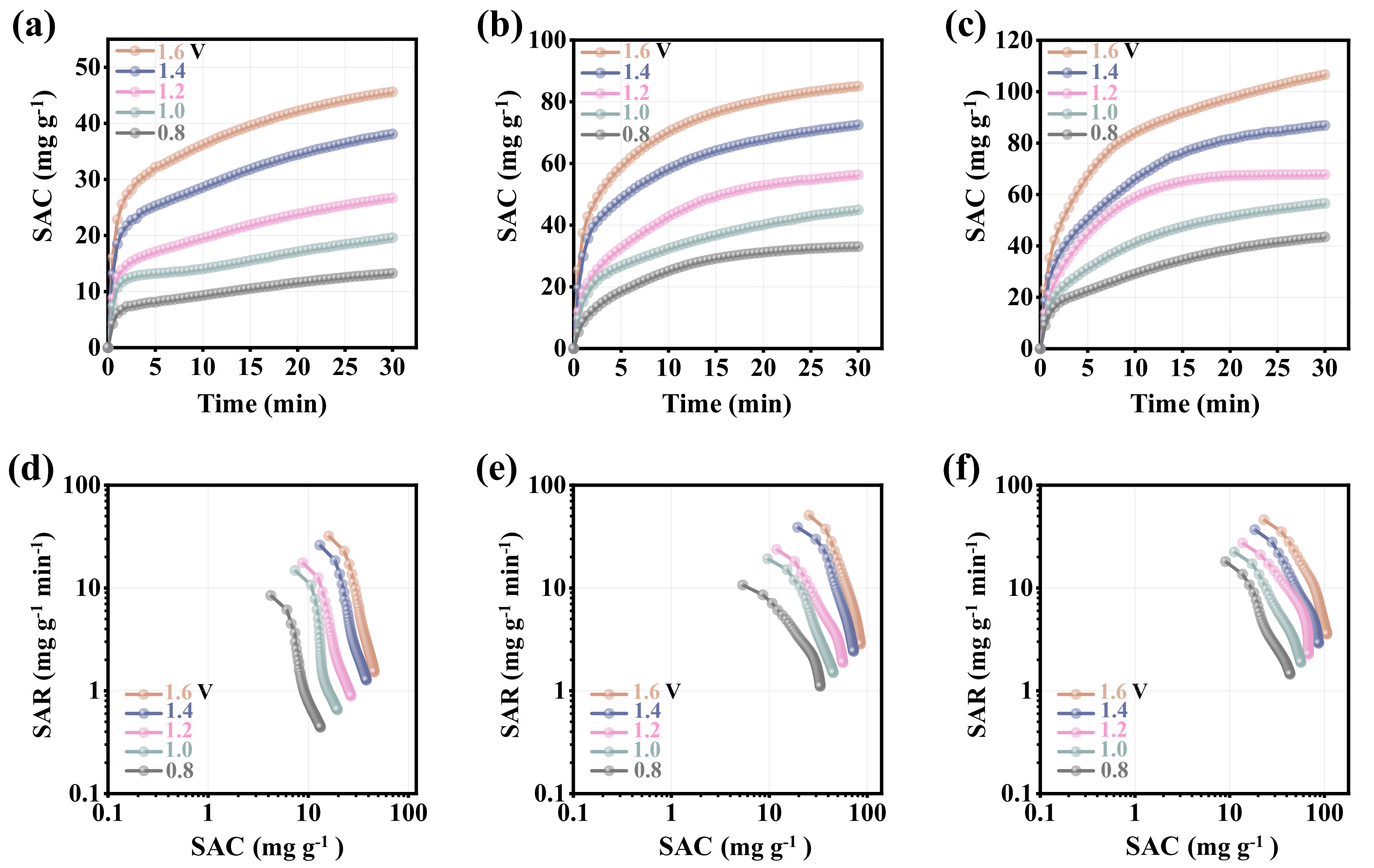

Fig. S9. SAC versus deionization time plots of the (a) ZIF-8-C, (b) NC/MoC-0.25, and (c) NC/MoC/Mo_2C-1 electrodes with initial concentration of 500 mg L^{-1} using different voltages. The corresponding CDI Ragone plots for (d) ZIF-8-C, (e) NC/MoC-0.25, and (f) NC/MoC/Mo_2C-1.

Table S1. Comparisons of the transition metal carbides/dichalcogenides or MOFs-derived carbons for their CDI performance.

**Samples**[a]	**SSAs** (m^2 g^{-1})	**NaCl concentrations** (mg L^{-1})	**Voltages** (V)	**SACs** (mg g^{-1})	**Refs.**
MoC@CNFAs	351.4	3000	1.2	37.03	[S16]
WC@GNFs	379	150 (300 μS m^{-1})	1.4	22.155	[S17]
$CoCr_7C_3$@CNFs	-	500 (1000 μS m^{-1})	1.0	20.4	[S18]
T-MoS_2	42.8	100	0.8	35.0	[S19]
MoS_2-S180	-	500	0.8	28.85	[S20]
MoS_2/CNT	225	300	0.8	10	[S21]
MoS_2/PDA	-	200	1.2	16.94	[S22]
MoS_2-rGO	28.7	200	1.0	16.82	[S23]
MoS_2/g-C_3N_4	174.3	250	1.6	24.16	[S24]
MoS_2/NOMC	233.4	250	1.6	28.82	[S25]
Ce-MoS_2	3.71	400	1.2	8.81	[S26]
NC@GC/CNTs	516.46	1000	1.6	56.30	[S27]
NPC/rGO	1336	1000	1.2	39.34	[S28]
CNF_{ZIF}	416	500	1.2	50.88	[S29]
NHPC	848	500	1.4	20.05	[S30]
NC-800	798	50	1.2	8.52	[S31]
NP-EHPC	1165.8	500	1.2	24.14	[S32]
P-CNFA	728.2	1000	1.2	16.20	[S33]
NC/MoC-0.5	506.3	500	1.2	84.2	This study
		1000	1.6	123.4	

[a] **MoC@CNFAs**: Molybdenum carbide nanoparticle-embedded carbon nanofiber aerogels; **WC@GNFs**: Tungsten carbide@graphene nanoflakes; **$CoCr_7C_3$@CNFs**: $CoCr_7C_3$ metallicemetallic carbides@carbon nanofibers; **T-MoS_2**: Defect-rich MoS_2; **MoS_2-S180**: Oxygen-incorporated MoS_2 that synthesized at 180 ºC; **MoS_2-CNT**: Molybdenum disulfide /carbon nanotube; **MoS_2/PDA**: MoS_2/polydopamine; **MoS_2-rGO**: 3D flower-like MoS_2/reduced graphene oxide composite; **MoS_2/g-C_3N_4**: MoS_2/graphitic carbon nitride composite; **MoS_2/NOMC**: MoS_2/nitrogen-doped highly ordered mesoporous carbon; **Ce-MoS_2**: Chemically exfoliated multi-layer MoS_2 nanosheets; **NC@GC/CNTs**: Nitrogen-doped carbon@graphitic carbon/carbon nanotubes; **NPC/rGO**: Porous N, P-doped carbon/reduced graphene oxide; **CNF_{ZIF}**: Carbon nanofiber@ZIF-8 derived N-rich porous carbon nanosheets; **NHPC**: Hierarchical porous ZIF-8 derived carbon; **NC-800**: ZIF-8 derived N-doped carbon by the pyrolysis at 800 ºC; **NP-EHPC**: N, P co-doped eave-like hierarchical porous carbon; **P-CNFA**: P-doped carbon nanofiber aerogels.

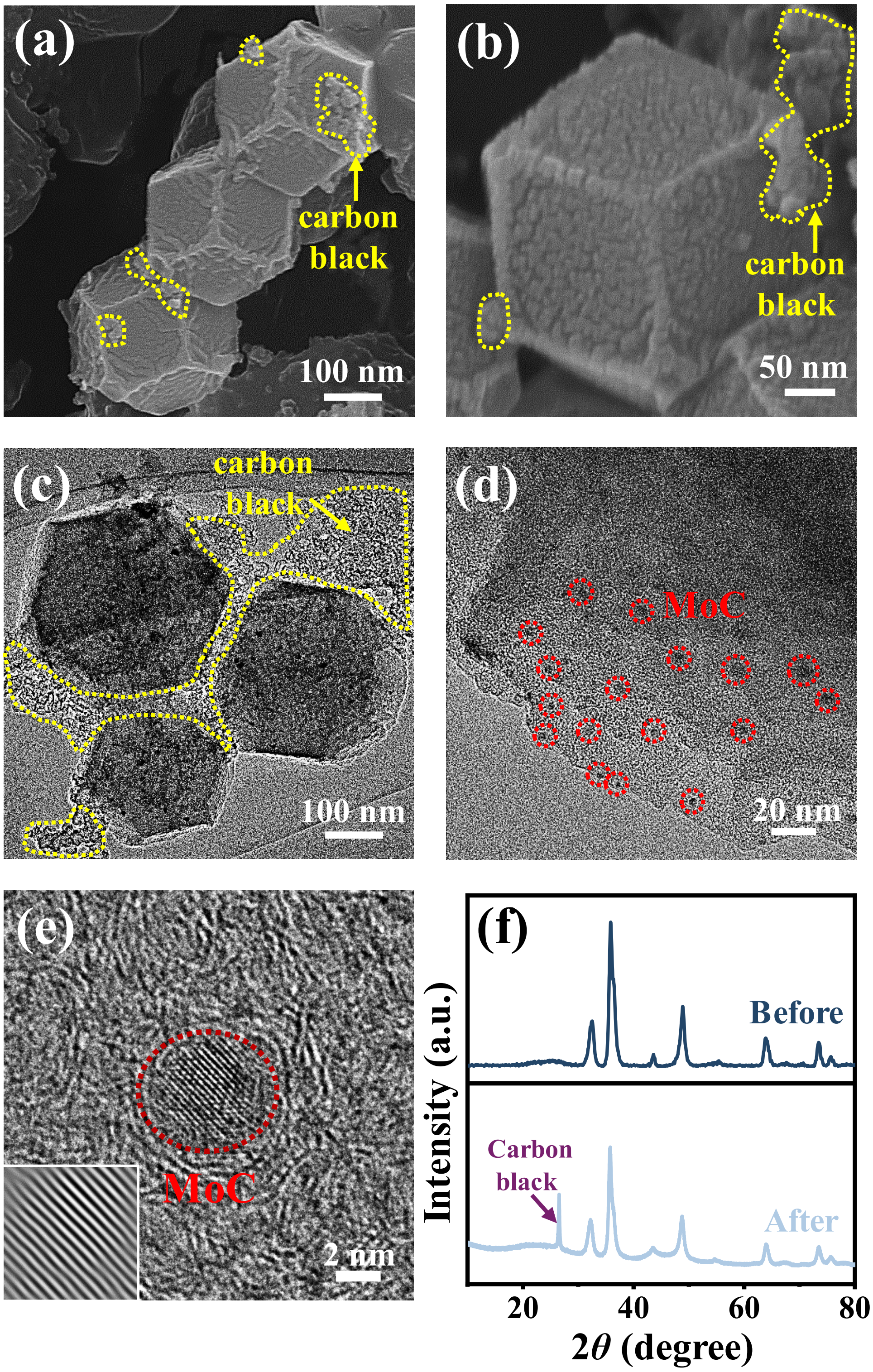

Fig. S10. (a, b) SEM, (c, d) TEM, (e) HRTEM (Insert: the IFFT image), and (f) XRD pattern of NC/MoC-0.5 after the 200 CDI cycles (Applied voltage: 1.2 V; NaCl concentration: 500 mg L^{-1}).

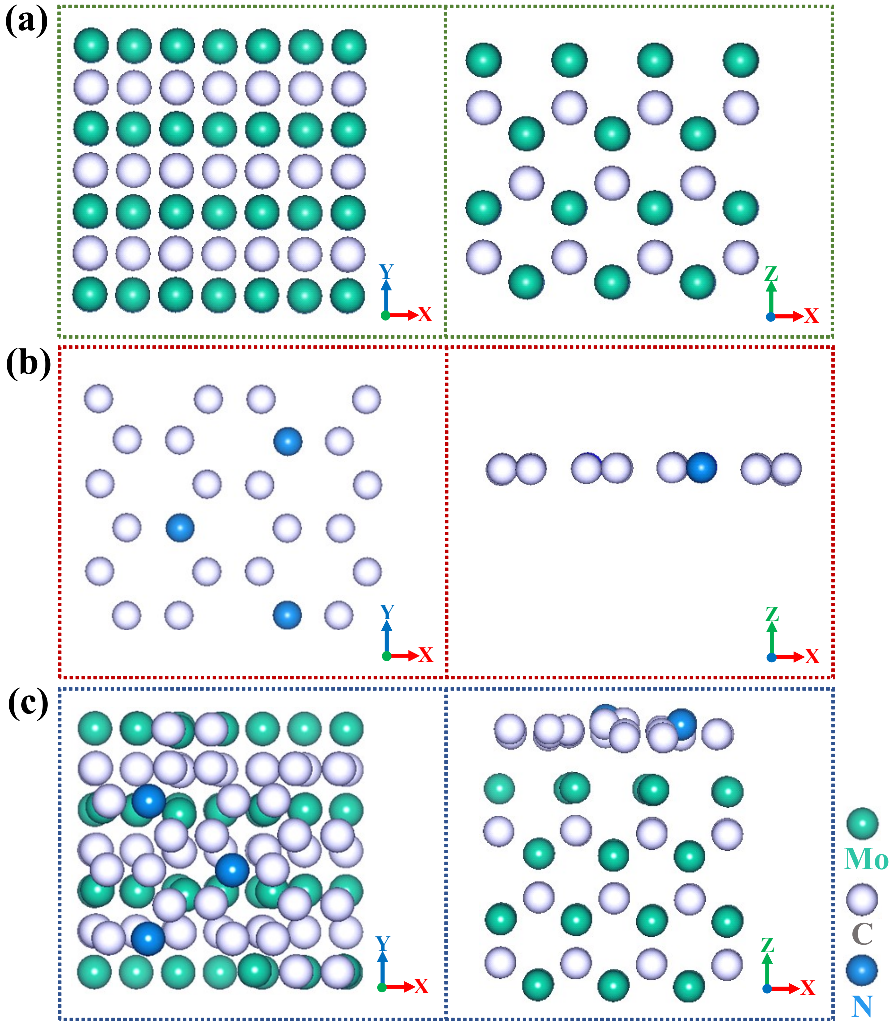

Fig. S11. The structure models of (a) MoC, (b) NC, and (c) NC/MoC [S34, S35].

References

[S1] X. F. Lu, L. Yu, J. Zhang, X.-W. Lou, *Adv. Mater.*, 2019, **31**, 1900699.

[S2] C. Avci, J. Ariñez-Soriano, A. Carné-Sánchez, V. Guillerm, C. Carbonell, I. Imaz, D. Maspoch, *Angew. Chem. Int. Ed.*, 2015, **54**, 14417.

[S3] Z. Yang, T. Zhao, X. Huang, X. Chu, T. Tang, Y. Ju, Q. Wang, Y. Hou, S. Gao, *Chem. Sci.*, 2017, **8**, 473-481.

[S4] Y. Li, N. Chen, Z. Li, H. Shao, X. Sun, F. Liu, X. Liu, Q. Guo, L. Qu, *Adv. Mater.*, 2021, **33**, 2105853.

[S5] T. Yang, H. Zhang, L. Guo, J. Wang, Z. Guo, Y. Du, J. Liu, Y. Zhao, P. Zhang, Z.-Y. Ji, *Desalination* 2023, **567**, 116984.

[S6] Y. Zhang, H. Li, Q. Yang, S. Zhang, B. Zhao, J. Wu, N. Shang, X. Zhao, Z. Xiao, X. Zang, J. Kim, X. Xu, Y. Yamauchi, *J. Mater. Chem. A* 2023, **11**, 14356.

[S7] S. Wang, Z. Li, G. Wang, Y. Wang, Z. Ling, C. Li, *ACS Nano* 2021, **16**, 1239-1249.

[S8] J. Zhao, B. Wu, X. Huang, Y. Sun, Z. Zhao, M. Ye, X. Wen, *Adv. Sci.*, 2022, **9**, 2201678.

[S9] X. Xu, S. Zhang, J. Tang, L. Pan, M. Eguchi, J. Na, Y. Yamauchi, *EnergyChem* 2020, **2**, 100043.

[S10] S. Cao, Y. Li, Y. Tang, Y. Sun, W. Li, X. Guo, F. Yang, G. Zhang, H. Zhou, Z. Liu, Q. Li, M. Shakouri, H. Pang, *Adv. Mater.*, 2023, **35**, 2301011.

[S11] S. Gong, H. Liu, F. Zhao, Y. Zhang, H. Xu, M. Li, J. Qi, H. Wang, C. Li, W. Peng, X. Fan, J. Liu, *ACS Nano* 2023, **17**, 4843-4853.

[S12] J. Wu, X. Xuan, S. Zhang, Z. Li, H. Li, B. Zhao, H. Ye, Z. Xiao, X. Zhao, X. Xu, X. Liu, J. You, Y. Yamauchi, *Chem. Eng. J.*, 2023, **473**, 145421.

[S13] W. Kong, X. Ge, Q. Zhang, F. Zhang, D. Kong, X. Zhu, *Carbon* 2023, **215**, 118501.

[S14] H. Arif, M. B. Tahir, B. S. Almutairi, I. Khalid, M. Sagir, H. Elhosiny Ali, H. Alrobei, M. Alzaid, *Inorg. Chem. Commun.*, 2023, **150**, 110474.

[S15] M. D. Segall, J. D. L. Philip, M. J. Probert, C. J. Pickard, P. J. Hasnip, S. J. Clark, M. C. Payne, *J. Phys.: Condens. Matter.*, 2002, **14**, 2717.

[S16] Y. Liu, Y. Zhang, Y. Zhang, Q. Zhang, X. Gao, X. Dou, H. Zhu, X. Yuan, L. Pan, *J. Mater. Chem. A* 2020, **8**, 1443-1450.

[S17] A.M. Al-Enizi, R.M.A. Hameed, M.M. El-Halwany, M. Bakrey, S.F. Shaikh, A. Yousef, *J. Colloid Interf. Sci.*, 2021, **581**, 112-125.

[S18] N. Zouli, R.M.A. Hameed, A. Abutaleb, M.M. El-Halwany, M.H. El-Newehy, A. Yousef, *J. Mater. Res. Technol.*, 2021, **15**, 3795-3806.

[S19] F. Jia, K. Sun, B. Yang, X. Zhang, Q. Wang, S. Song, *Desalination* 2018, **446**, 21-30.

[S20] K. Sun, X. Yao, B. Yang, F. Jia, S. Song, *Desalination* 2020, **496**, 114758.

[S21] P. Srimuk, J. Lee, S. Fleischmann, S. Choudhury, N. Jäckel, M. Zeiger, C. Kim, M. Aslan, V. Presser, *J. Mater. Chem. A* 2017, **5**, 15640-15649.

[S22] Q. Wang, F. Jia, S. Song, Y. Li, *Sep. Purif. Technol*., 2020, **236**, 116298.

[S23] W. Peng, W. Wang, G. Han, Y. Huang, Y. Zhang, *Desalination* 2020, **473**, 114191.

[S24] S. Tian, X. Zhang, Z. Zhang, *Desalination* 2020, **479**, 114348.

[S25] S. Tian, X. Zhang, Z. Zhang, *Chem. Eng. J.*, 2021, **409**, 128200.

[S26] F. Xing, T. Li, J. Li, H. Zhu, N. Wang, X. Cao, *Nano Energy* 2017, **31**, 590-595.

[S27] Y. Zhang, J. Wu, S. Zhang, N. Shang, X. Zhao, S.M. Alshehri, T. Ahamad, Y. Yamauchi, X. Xu, Y. Bando, *Nano Energy* 2022, **97**, 107146.

[S28] J. Guo, X. Xu, J.P. Hill, L. Wang, J. Dang, Y. Kang, Y. Li, W. Guan, Y. Yamauchi, *Chem. Sci.*, 2021, **12**, 10334-10340.

[S29] X. Gong, W. Luo, N. Guo, S. Zhang, L. Wang, D. Jia, L. Ai, S. Feng, *J. Mater. Chem. A* 2021, **9**, 18604-18613.

[S30] Z. Wang, T. Yan, L. Shi, D. Zhang, *ACS Appl. Mater. Interfaces* 2017, **9**, 15068-15078.

[S31] N.-L. Liu, S. Dutta, R.R. Salunkhe, T. Ahamad, S.M. Alshehri, Y. Yamauchi, C.-H. Hou, K.C.W. Wu, *Sci. Rep.*, 2016, **6**, 28847.

[S32] H. Zhang, C. Wang, W. Zhang, M. Zhang, J. Qi, J. Qian, X. Sun, B. Yuliarto, J. Na, T. Park, H.G.A. Gomaa, Y.V. Kaneti, J.W. Yi, Y. Yamauchi, J. Li, *J. Mater. Chem. A* 2021, **9**, 12807-12817.

[S33] Y. Li, Y. Liu, M. Wang, X. Xu, T. Lu, C.Q. Sun, L. Pan, *Carbon* 2018, **130**, 377-383.

[S34] J. Qiu, Z. Yang, Q. Li, Y. Li, X. Wu, C. Qi, Q. Qiao, *J. Mater. Chem. A* 2016, **4**, 13296-13306.

[S35] M. Yuan, H. Lv, H. Cheng, B. Zhao, G. Chen, J. Zhang, R. Che, *Adv. Funct. Mater.*, 2023, **33**, 2302003.
